# The Diminishing Effect of Transformational Leadership on the Relationship Between Task Characteristics, Perceived Meaningfulness, and Work Engagement

**DOI:** 10.3389/fpsyg.2020.585031

**Published:** 2020-11-25

**Authors:** Fanxing Meng, Yi Wang, Wenying Xu, Junhui Ye, Lin Peng, Peng Gao

**Affiliations:** ^1^Zhejiang Police College, Hangzhou, China; ^2^School of Marxism, Zhejiang Sci-Tech University, Hangzhou, China; ^3^Department of Psychology, Sun Yat-sen University, Guangzhou, China; ^4^Department of Pharmacy, The Children’s Hospital, Zhejiang University School of Medicine, National Clinical Research Center for Child Health, Hangzhou, China

**Keywords:** task autonomy, task significance, transformational leadership, meaningfulness in work, work engagement

## Abstract

The topic of employee work engagement in the public sector has attracted broad attention because it is critical to the efficiency and effectiveness of public services. Based on the Job Characteristics Model (JCM) and the Integrative Theory of Employee Engagement (ITEE), the present research adopts a multilevel design to examine a moderated mediation model in which task characteristics (i.e., task autonomy and task significance as level-1 predictors) and social context (i.e., transformational leadership as a level-2 moderator) jointly impact employee work engagement *via* individual perception of meaningfulness in work. A total of 349 grassroots police officers from 35 police substations were invited to anonymously complete a survey *via* mobile app. After performing the cross-sectional analysis, the results indicated that in contrast to task significance, the conditional effect of task autonomy on work engagement *via* perceived meaningfulness was more positive at a lower level of transformational leadership. Implications, limitations, and future research directions are discussed.

## Introduction

Over the past 2 decades, the number of studies on work engagement, which is regarded as a more robust predictor of work performance than other factors such as job satisfaction and commitment (Bakker, 2015), has increased rapidly (Bakker and Albrecht, 2018). In the public sector, employees’ work engagement is very important because its outcome influences people’s experience and evaluation of and their satisfaction with public services (Harter et al., 2002; Pritchard, 2008). However, public-sector employees are likely to be less engaged in the organizations they work for compared to employees in the private sector (Agyemang and Ofei, 2013). It is possible that this trend may be closely related to the inherent characteristics of public sectors that can create barriers to engagement (Lavigna, 2015), such as the patterns of leadership and decision making (Pollitt and Bouckaert, 2011).

The present research proposes and examines a conditional model to reveal the underlying mechanism by which inherent characteristics in the working environment of public sectors impact the work engagement of employees. According to the theory of purposeful work behavior (Barrick et al., 2013), vital motivational attributes of the work environment can be described through two major components: task characteristics and social context. Jobs that provide task characteristics such as task autonomy and task significance are more motivating (Fried and Ferris, 1987). Similarly, jobs with a positive social context such as transformational leadership, provide more opportunities for employees to acquire strong interpersonal support and encouragement (Grant and Parker, 2009).

The motivational attributes of both task characteristics (e.g., May et al., 2004) and leadership (e.g., Bono and Judge, 2003) are likely to activate relevant psychological processes and further impact employees’ degree of engagement. Task characteristics can arouse meaningful, valuable, and worthwhile feelings in employees about their work (Hackman and Oldham, 1975), which in turn facilitates their engagement (May et al., 2004). That is, task characteristics are likely to relate to the work engagement of employees in public sectors through personal meaningful experience. Transformational leadership, which typically encourages goal-striving behavior (Hamstra et al., 2011), can enable followers to experience meaningfulness in their work, which in turn facilitates their engagement (Barrick et al., 2013; Saks and Gruman, 2014). Transformational leadership is likely to play a moderating role in the mediating process in which task characteristics relate to the work engagement of employees through perceived meaningfulness.

This article aims to contribute to the dearth of research on the joint effects of task characteristics and social context (i.e., leadership) in the engagement process by theorizing and examining (a) a mediating process of perceived meaningfulness in which task characteristics drive employees’ work engagement and (b) a moderating role of transformational leadership on the mediating process of meaningfulness. In addition, this research contributes to the practice of primary-level management by applying and adjusting (a) the skills of transformational leaders with regard to the characteristics of the task and (b) the work redesign of specific tasks to activate individual meaningful experiences.

## Theoretical Foundations and Hypothesis Development

Work engagement is a positive psychological state of employees that is reflected in task-related behavior. Kahn (1990) first introduced work engagement academically as the employment and expression of the self in task behaviors that promote connections to work and to others, personal presence (physical, cognitive, and emotional), and active, full role performances. Kahn’s definition of work engagement indicates that the engagement of employees is obvious while they are working on a task. Schaufeli and Bakker (2004) subsequently described work engagement as a work-related state of mind that is characterized by vigor (energy and mental resilience in work), dedication (high enthusiasm and involvement in work), and absorption (full concentration in work). Employees show their vigor, dedication, and absorption when they are willing to become involved in and address a task.

Task characteristics are important antecedents of work engagement (Bakker et al., 2014; Saks, 2019). Usually, various tasks are clustered together to make up a job (Ilgen and Hollenbeck, 1991). The between-task variability of engagement during the working process is not random but is closely bonded to specific characteristics of the task (Sonnentag, 2017). The Job Characteristics Model (JCM; Hackman and Oldham, 1975) specifies core task characteristics that stimulate critical psychological states and influence personal work outcomes. In the job demands-resources (JD-R) model, task characteristics, including task autonomy and significance, are regarded as important job resources to activate a motivational pathway leading to work engagement at the task level (Bakker and Demerouti, 2007), because they fulfill basic human needs according to Self-Determination Theory, such as the needs for autonomy, competence, and relatedness (Deci and Ryan, 1985). The present research concentrates on two task characteristics that have received considerable research attention and are highly embodied in police officers’ work: task autonomy and task significance. For grassroots police officers, task significance and task autonomy are usually reflected directly before the task begins or in progress, unlike the reflection of feedback from job, task identity, and skill variety in JCM after task completion. Due to the assignment of numerous temporary tasks and the indefinite duration of evidence investigation and judicial procedures, grassroots police officers are often deployed to perform other tasks before the previous task is completed and closed. Accordingly, task autonomy and task significance are inclined to be more certain and distinct, compared with feedback from job, task identity, and skill variety. Task autonomy refers to the extent to which a job provides discretion over routine work decisions, such as when and how to perform a task (Parker, 2014). On the basis of Job Demands-Control Model (Karasek, 1979), the combination of low decision latitude and heavy job demands is associated with mental strain and job dissatisfaction. A highly autonomous task is likely to encourage employees to apply different work approaches and methods (De Spiegelaere et al., 2014) and improve the efficiency and effectiveness of their work completion. Task significance refers to the extent of the impact a job has on the lives or work of others (Hackman and Oldham, 1975). Highly significant tasks are likely to strengthen employees’ perception of the impact of their work on others and the feeling of being valued by the beneficiaries; these tasks thus have a positive effect on employees’ engagement (Goštautaitė and Bučiūnienė, 2015). Previous studies have confirmed that work engagement could be positively predicted by task autonomy (e.g., Quiñones et al., 2013) and task significance (e.g., Goštautaitė and Bučiūnienė, 2015). Experiencing meaningfulness in work plays a critical role in the relationship between task characteristics and work engagement. According to the JCM, task characteristics influence work outcomes (e.g., work engagement) through critical psychological conditions (Hackman and Oldham, 1975; Kahn, 1990). Meta-analysis results indicate that compared to experienced responsibility and knowledge of results, experienced meaningfulness is the “most critical” psychological state (Humphrey et al., 2007). The Integrative Theory of Employee Engagement (ITEE; Saks and Gruman, 2014) suggests that task characteristics, such as task autonomy and task significance, correspond with experiencing meaningfulness in work and subsequent work engagement on the task level. Perceived meaningfulness in work is likely to be a mediator between task characteristics and work engagement. Based on the identity perspective, Pratt and Ashforth (2003) distinguish the meaningful work experience into meaningfulness in work and at work. Meaningfulness in work is about the roles in work (e.g., tasks) and can be used to explain the question: “what am I doing?” Meaningfulness at work is about the membership and can be used to explain the question: “where do I belong?” Conceptually, meaningfulness in work refers to intrinsic motivation while an employee conducts a task; it differs from meaningfulness at work, which focuses on the motivating effect of the membership function as an employee performs his/her role according to organizational goals, values, and beliefs (Stein et al., 2018). In fact, experiencing personal meaning in work is closely related to higher-level needs for achievement and self-actualization on Maslow’s hierarchy and to maximizing one’s sense of motivation in work and fulfilling one’s life purpose (Ghadi et al., 2013). A lack of meaningfulness in an employee’s work can be closely associated with work alienation and dissonance (Bailey et al., 2017).

*Hypothesis 1:* Perceived meaningfulness in work mediates the relationship between task autonomy and work engagement.
*Hypothesis 2:* Perceived meaningfulness in work mediates the relationship between task significance and work engagement.

The social context (e.g., leadership) could translate task characteristics (i.e., task autonomy and task significance) into work engagement *via* perceived meaningfulness. Previous literature shows that task characteristics and social context are mutually independent components of the work environment (Hu et al., 2016) and can be bound together to influence positive individual outcomes, for example, creative work involvement (Volmer et al., 2012). As a typical social context, transformational leadership is an exclusive style of leadership in which a leader works with teams to identify areas of improvement, creates a vision to guide change through inspiration, and executes the change in tandem with committed members of a group (Bass, 1999). A wealth of evidence shows that transformational leadership behaviors are effective in a range of organizational processes (e.g., Podsakoff et al., 1990; Bass and Riggio, 2006; Nielsen, 2013). The effectiveness of transformational leadership operates more at the team or organizational level (Parry, 2011). Followers in a working team tend to be influenced by transformational leaders through underlining the similarity within members of a team, modeling collective commitment, and strengthening collective goals, shared values, and common interests (Shamir et al., 1993; van Knippenberg et al., 2004). However, the negative effects of transformational leadership have gradually emerged with the deepening of research. Barnett et al. (2001) took a sample of secondary school teachers and found that the transformational leadership behavior (vision/inspiration) was negatively linked to student learning culture. Teachers may be distracted from focusing on learning and teaching to take part in the pastoral care and many other school-level activities that are regarded as important for achieving and gaining teacher support for the vision of the school. In the study of Jiang et al. (2015), they found that individual-focused transformational leadership was negatively linked to team innovation *via* team knowledge sharing in a sample from four firms in the high-tech industry. Individual-focused transformational leadership may impede the formation of a common goal of team and the sharing of knowledge and constructive feedback of members in the team. Li and Yuan (2017) took a sample from eight four- and five-star hotels and found that transformational leadership has indirect negative moderation effect on the relationship between proactive personality and career satisfaction through its influence on leader–leader exchange. The transformational supervisors may encounter poor-quality leader–leader exchange of hotel employees because of their dark side like being boastful, self-centered, and rapt in superiority and power (Bass, 1998; Rosenthal and Pittinsky, 2006).

**Figure 1:**
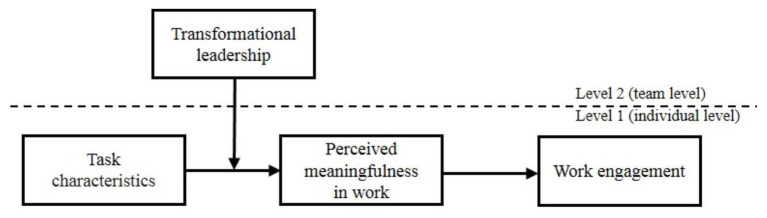
Proposed moderated mediation model.

We expect that transformational leadership can diminish the positive indirect effect of perceived meaningfulness, although it has been verified that transformational leadership positively relates to followers’ meaningful perception (e.g., Frieder et al., 2017) and work engagement (e.g., Ghadi et al., 2013; Espinoza-Parra et al., 2015). Transformational leadership is likely to constrain the extent to which relatively stable task characteristics can influence the perception of the meaningfulness of work. Meaningfulness in work can be facilitated by organizational practices that enrich the tasks and working roles that an individual performs (Pratt and Ashforth, 2003) and that influence relevant work engagement (May et al., 2004). That is, it is feasible that the multilevel analysis can be conducted to study the effect of transformational leadership from the team level on individual perceived meaningfulness in work. When the degree of transformational leadership is high, it is more likely for police officers to be stimulated by their immediate superior to think about and execute changes to the status quo and make progress. Police officers tend to feel stressed due to extra requirements, increased workload and the risk of violating existing operating rules from transformational leaders, and hardly to perceive meaningfulness of work. That is, the effect of task characteristics (i.e., task autonomy and task significance) is weakened in a strong context of transformational leadership. Specifically, with regard to task autonomy, employees with high autonomy in routine work gain an increased sense of meaningfulness (Kiggundu, 1983). To encourage followers to strive for implicit higher-order achievement, transformational leaders provide clear guidance and strong norm-setting for followers regarding anticipated work attitudes and behavior (Bass, 1985), which are likely to limit perceived meaningfulness for those working on tasks that support autonomy. With regard to task significance, transformational leaders infuse a heightened understanding into the contextual details of work by providing a compelling vision for their followers, aligning specific expectations with individuals, establishing collective goals (Frieder et al., 2017), and inspiring employees to explore new approaches to problem-solving (Bass, 1999). Transformational leadership, as a strong social context of work, tends to overemphasize the value of work beyond the sense of meaningfulness of the task itself. Accordingly, it can be assumed that strong transformational leadership as a social context may weaken the association between police officers’ task characteristics and their perceived meaningfulness. Furthermore, strong transformational leadership indirectly weakens the relationship between police officers’ task characteristics and relevant work engagement (see Figure 1).

*Hypothesis 3:* Transformational leadership moderates the indirect effect of task autonomy on engagement through perceived meaningfulness such that the indirect effect will be less (more) positive when transformational leadership is high (low).
*Hypothesis 4:* Transformational leadership moderates the indirect effect of task significance on engagement through perceived meaningfulness such that the indirect effect will be less (more) positive when transformational leadership is high (low).

## Materials and Methods

### Participants

Serving people wholeheartedly is the core requirement for Chinese police (Wang and Wong, 2012). The police work in China combines the unique features of challenging, stressful tasks, high risk, and frequent unexpected events (Lan et al., 2020). In 2019, 6,211 police officers and 5,699 auxiliary police officers were injured on duty, and 280 police officers and 147 auxiliary police officers died on duty (data from the website of the Ministry of Public Security of the People’s Republic of China). Chinese police have strict work regulations and a heavy workload (Lan et al., 2020). It is common for them to work overtime to tackle with various emergencies (Liu et al., 2019), for example, working with medical workers together to prevent coronavirus disease 2019 (COVID-19).

Police officers as participants were recruited from a training program in turns at the police college. The contents and teachers were the same in each round of training. In general, one to four police officers from the same police substation participated in each training. The data were collected by taking each police substation as a cluster unit. In order to avoid the situations of temporary non-participation in training or unwillingness to participate in this study, the total attending number of a police substation was required for greater than or equal to 12. Thirty-five police substations were randomly selected. Participants received and completed the survey through the mobile app after obtaining their positive permission. After removing an invalid data, 349 data in total were as the sample of this study. That is, 10 participants per police substation were recruited from 34 police substations. Nine participants were from another police substation. The final sample was 92% male and 8% female. The participants’ age ranges were 20–30 years (17.8%), 30–40 years (32.7%), 40–50 years (27.8%), and above 50 years (21.7%). Years of service were 1–5 years (14.3%), 6–10 years (19.2%), 11–20 years (33.2%), 21–30 years (20.1%), and above 30 years (13.2%).

### Measures

The survey in the present research contained five parts. Participants were asked to rate their level of agreement with each statement on a five-point Likert scale.

Task autonomy: Autonomy was assessed using four items (Cronbach’s *α* = 0.733, see Table 1) adapted from Job Autonomy Scale of Beehr (1976) and instrument of Peccei and Rosenthal (2001). A sample item was “I can make decisions on my own in my work.”

**Table 1:** Descriptive statistics and correlation coefficient^a^.

	*M*	*SD*	1	2	3	4	5
1. Autonomy	3.686	0.758	(0.733)				
2. Significance	3.265	0.825	0.201^*^	(0.827)			
3. Transformational leadership	3.493	0.972	0.198^*^	0.135^*^	(0.913)		
4. Perceived meaningfulness	3.766	0.799	0.599^*^	0.293^*^	0.411^*^	(0.799)	
5. Work engagement	3.505	0.751	0.496^*^	0.25^*^	0.36^*^	0.557^*^	(0.738)

a Internal consistency reliabilities are reported in parentheses along the diagonal.

* *p* < 0.05.

Task significance: Task significance was measured using four items (*α* = 0.827) adapted from measure of Grant (2008). A sample item was “My job enhances the wellbeing of other people.”

Transformational leadership: Transformational leadership (*α* = 0.913) was assessed using a seven-item short measure of transformational leadership (Carless et al., 2000). A sample item was “Communicates a clear and positive vision of the future.”

Perceived meaningfulness in work: Perceived meaningfulness (*α* = 0.799) was operationalized using the four-item scale adapted from May et al. (2004) and Bunderson and Thompson (2009). A sample item was “I feel that the work I do on my job is valuable.”

Work engagement: Work engagement (*α* = 0.738) was assessed using the ultra-short measure for work engagement (Schaufeli et al., 2019). A sample item was “In my work, I am bursting with energy.”

### Analytical Strategy

Considering the nested feature of the data (i.e., police officers in the same police substation shared the same direct supervisor), multilevel modeling (MLM) was employed to analyze the data of the present research. The hypothetical model was tested with the MLmed Beta 2 macro for SPSS software (Hayes and Rockwood, 2020). Utilizing this analytic approach, a multilevel moderated mediation model was proposed. It estimated (see Figure 1) transformational leadership as a level-2 moderator of the level-1 indirect effect of task resources (i.e., autonomy and significance) on work engagement *via* perceived meaningfulness in work. Before the formal analysis, the intraclass correlation (ICC) values of the mediator and dependent variable were calculated to estimate the dependence magnitude. The results showed that group membership within the same police substation accounted for more than 30% of convergence in perceived meaningfulness (*ICC* = 0.383) and work engagement (*ICC* = 0.353). Similar to classroom-based clustering, ICC values of approximately 0.3 are common for police substations (Musca et al., 2011).

## Results

The confirmatory factor analysis (CFA) with all five correlated constructs was analyzed using AMOS 21.0 software and provided a good fit to the data, *χ^2^*/*df* = 1.378 (<5), GFI = 0.934 (>0.9), TLI = 0.975 (>0.9), CFI = 0.978 (>0.9), and RMSEA = 0.033 (<0.08). The result of Harman’s single factor test on all items indicated that the first factor accounted for 36.318% (<40%) of total variance.

**Figure 2:**
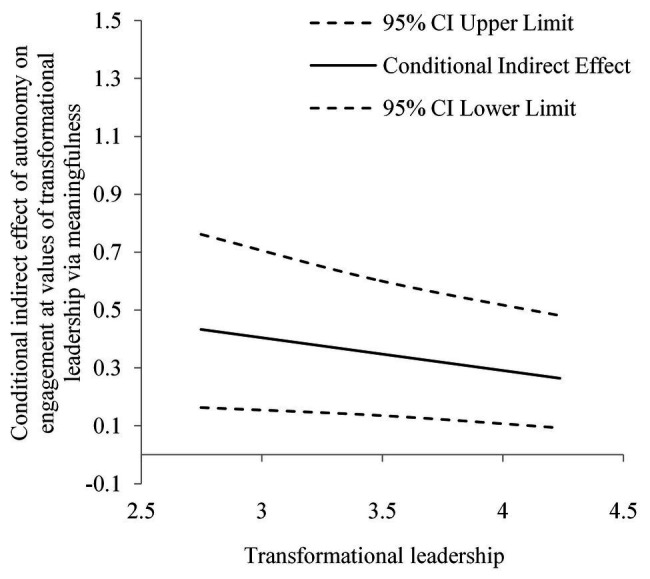
Moderated mediation plot of Hypothesis 3.

Descriptive statistics and correlations between the variables are shown in Table 1. Mean scores of variables range between 3.265 and 3.766. The significant coefficients of pairwise correlation range between 0.198 and 0.599. The results of the moderated mediation analysis are reported in Tables 2 and 3. As shown in Table 2, the indirect effect of autonomy on engagement *via* meaningfulness was significant [*effect* = 0.745, 95% CI (0.222, 1.443)]. A significant and negative interaction effect was found in which transformative leadership moderated the link between autonomy and meaningfulness [*b* = −0.249, 95% CI (−0.494, −0.005)]. The index of moderated mediation was significant and negative [*effect* = −0.113, 95% CI (−0.259, −0.005)]. The conditional indirect effect of autonomy on engagement at a low level of transformational leadership [*effect* = 0.433, 95% CI (0.164, 0.762)] was greater than the effect at a high level of transformational leadership [*effect* = 0.264, 95% CI (0.093, 0.481)]. Hypotheses 1 and 3 were supported (see Figure 2).

**Table 2:** Testing results of Hypotheses 1 and 3^a^.

	Meaningfulness				Engagement			
	*b*	*SE*	*LL*	*UL*	*b*	*SE*	*LL*	*UL*
Autonomy	1.636	0.477	0.664	2.61	0.551	0.186	0.171	0.931
TFL	1.321	0.458	0.387	2.254				
Autonomy × TFL	−0.249	0.12	−0.494	−0.005				
Meaningfulness					0.455	0.132	0.186	0.724
					***Effect***	***SE***	***MCLL***	***MCUL***
Indirect effect					0.745	0.312	0.222	1.443
**Conditional indirect effect at:**								
Low TFL (−1 *SD*)					0.433	0.152	0.164	0.762
High TFL (+1 *SD*)					0.264	0.099	0.093	0.481
Index of moderated mediation					−0.113		−0.259	−0.005

a TFL is short for transformational leadership.

**Table 3:** Testing results of Hypotheses 2 and 4^a^.

	Meaningfulness				Engagement			
	*b*	*SE*	*LL*	*UL*	*b*	*SE*	*LL*	*UL*
Significance	0.005	0.642	−1.305	1.314	0.093	0.119	−0.148	0.334
TFL	0.474	0.589	−0.727	1.674				
Significance × TFL	0.031	0.172	−0.321	0.382				
Meaningfulness					0.749	0.09	0.565	0.933
					***Effect***	***SE***	***MCLL***	***MCUL***
Indirect effect					0.004	0.485	−0.95	0.965
**Conditional indirect effect at:**								
Low TFL (−1 *SD*)					0.067	0.155	−0.232	0.375
High TFL (+1 *SD*)					0.102	0.129	−0.143	0.36
Index of moderated mediation					0.023		−0.234	0.282

a TFL is short for transformational leadership.

As presented in Table 3, the results indicated that the indirect effect of significance on engagement *via* meaningfulness was not significant [*effect* = 0.004, 95% CI (−0.95, 0.965)]. The interaction between significance and transformational leadership was not significant [*b* = 0.031, 95% CI (−0.321, 0.382)]. The index of moderated mediation [*effect* = 0.023, 95% CI (−0.232, 0.279)] was not significant. The conditional indirect effects of significance on engagement *via* meaningfulness were not significant at the low level [*effect* = 0.067, 95% CI (−0.232, 0.375)] or the high level [*effect* = 0.102, 95% CI (−0.143, 0.36)] of transformational leadership behavior. Support for Hypotheses 2 and 4 was not found.

## Discussion

The present research provides valuable insights into how the interplay between task characteristics and transformational leadership influences police officers’ work engagement through perceived meaningfulness in work. Based on data from 349 grassroots police officers, we found support for the differences in the conditional indirect effect of task autonomy but not for task significance. The results indicated that psychological state (i.e., meaningfulness in work) acts as a critical mediator to promote individual engagement in the work environment of public sectors. Transformational leadership, as a social context construct that focuses on interpersonal relationships, played a diminishing role as the indirect effect of meaningfulness in work is high.

## Theoretical Implications

First, the mediating effect of perceived meaningfulness in work was further clarified and confirmed. The results of this research showed that meaningfulness in work was an effective mediator between task autonomy and work engagement but not for task significance. According to the Self-Determination Theory (Ryan and Deci, 2000), employees have an inner drive to satisfy their basic psychological needs for competence (i.e., feeling efficacy and mastery), autonomy (i.e., feeling volitional and self-endorsed), and relatedness (i.e., feeling of belonging and being cared for; Pedersen et al., 2019). Work meaningfulness fulfills fundamental psychological need of autonomy, and then leads to work engagement of police officers (Lee et al., 2017), because it represents the intrinsic motivational energy (Di Fabio, 2017) that encourages police officers to devote themselves to their work while carrying out their work. The mediating effect of meaningfulness in work between task significance and work engagement was not found. Based on an analogy with the fact, Warr’s vitamin model considers that vitamin plays an indispensable role for physical health up to certain standard; after attainment of that standard, increased vitamin intake can be detrimental (Wang and Lee, 2009). In accordance with the vitamin model of Warr (1987), overly high or low level of job characteristics is harmful for positive mental state of employees. When the significance of task is too high, police officers can feel stressful and exhausted of spending additional time and effort that weakens the experience of meaningfulness and work engagement (e.g., Schulze and Steyn, 2007; George et al., 2008). JCM considers experiencing meaningfulness as one of three important psychological conditions between task characteristics and individual work outcomes (Hackman and Oldham, 1975; Humphrey et al., 2007). JCM does not further subdivide the connotations of experiencing meaningfulness, such as meaningful experience when immersing oneself in a task and as a member in an organization. Subsequent ITEE theoretically proposes that perceived meaningfulness in work may have a mediating effect between task characteristics and individual outcomes because they are all task-level variables (Saks and Gruman, 2014). However, empirical evidence that emphasizes and examines the mediating effect of meaningfulness in work is scarce. The results of this study expand the theory of JCM to deeply understand the effect of experiencing meaningfulness and to verify the mediating role of perceived meaningfulness in work between task autonomy and work engagement for ITEE.

Second, this research provides a richer understanding of the role of transformational leadership. Numerous studies present compelling evidence that transformational leadership is an antecedent to positive individual- and organizational-level outcomes (Gooty et al., 2009; Mendelson et al., 2019). However, because of the two sides of transformational leadership (Kark et al., 2003), the efforts of transformational leaders do not always foster positive employee outcomes (Chen et al., 2018); this is especially true for police officers (Loftus, 2009; Cockcroft, 2014). This research found that perceived meaningfulness and engagement for grassroots police officers can be weakened by the combined effect of high task autonomy and high transformational leadership. This diminishing effect may occur because the higher autonomy in work and greater responsibility for change provided by transformational leaders can add additional burdens, such as increased expression of ideas, more allocations of tasks, high standards of performance (Chen et al., 2018), higher work stress (Spector et al., 1988), and greater challenges in balancing the implementation of changes and established statutes, which may further reduce meaningful perception and engagement in work. The results of this research can explain why employees’ work engagement in public sectors is insufficient from one perspective. With regard to the insignificant mediating and moderating effects of task significance, police officers may not be sensitive to the significance of routine tasks because almost all of their work matters as judicial workers, although they are inspired by transformative leaders in meaningful future visions. The results of this research further expand the theory of ITEE, in which the effect of transformational leadership can be not only an antecedent of task characteristics but also a moderator between task characteristics and perceived meaningfulness.

## Practical Implications

First, for primary-level management, police officers’ perceived meaningfulness in routine work should be attached to its importance. For example, grassroots police officers should be provided with adequate room to arrange and complete tasks in compliance with a rigid requirement framework of relevant laws and regulations, which can further strengthen meaningfulness perception and engagement facilitation.

Second, without violating laws and specific work regulations, basic-level leaders in police substations should tactically and tactfully apply transformational leadership skills to the management of grassroots police officers. For example, for police officers whose work context involves low task autonomy, transformational leaders could evoke a perception of heightened meaningfulness and consequently facilitate engagement by encouraging followers to take the initiative, challenge the status quo, adopt new strategies, and tolerate failure. The protective role of transformational leadership should attract enough attention in terms of perceived managerial support and subsequent organizational justice for police officers in low-autonomy tasks, which may significantly prevent the occurrence of counterproductive work behavior, such as unsafe behaviors, and workplace bullying (e.g., Guglielmi et al., 2018).

## Limitations and Implications for Future Research

Two limitations of the present research should be noted. First, we studied the mediating effect of meaningfulness in work but did not refer to meaningfulness at work. Perceived meaningfulness in work and at work should be examined and compared in the same model to reveal the mediating effect of intrinsic meaningful experience between the extrinsic work environment and individual work outcomes. Furthermore, we relied on employees’ self-report measurements on a single test. This research design was adopted because we were interested in the leader behavior and task features perceived by employees and in private experiences of meaningfulness and engagement. We encourage future studies to break through the restrictions of a single test in the present research and include both perceived meaningfulness in work and at work to validate the model proposed in this research by integrating longitudinal data or other-reported measurements.

## Data Availability Statement

The raw data supporting the conclusions of this article will be made available by the authors, without undue reservation.

## Ethics Statement

The studies involving human participants were reviewed and approved by Academic Committee of Zhejiang Police College. The patients/participants provided their written informed consent to participate in this study.

## Author Contributions

All authors listed have made a substantial, direct and intellectual contribution to the work, and approved it for publication.

### Conflict of Interest

The authors declare that the research was conducted in the absence of any commercial or financial relationships that could be construed as a potential conflict of interest.
